# Neuroimaging of Acute Intracerebral Hemorrhage

**DOI:** 10.3390/jcm10051086

**Published:** 2021-03-05

**Authors:** Peter B. Sporns, Marios-Nikos Psychogios, Grégoire Boulouis, Andreas Charidimou, Qi Li, Enrico Fainardi, Dar Dowlatshahi, Joshua N. Goldstein, Andrea Morotti

**Affiliations:** 1 Department of Neuroradiology, Clinic for Radiology & Nuclear Medicine, University Hospital Basel, 4031 Basel, Switzerland; Marios.psychogios@usb.ch; 2 Department of Diagnostic and Interventional Neuroradiology, University Medical Center Hamburg-Eppendorf, 20246 Hamburg, Germany; 3 Neuroradiology Department, University Hospital of Tours, CEDEX 09, 37044 Tours, France; g.boulouis@chu-tours.fr; 4 J. Philip Kistler Stroke Research Center, Department of Neurology, Massachusetts General Hospital, Harvard Medical School, Boston, MA 02114, USA; andreas.charidimou.09@ucl.ac.uk; 5 Department of Neurology, Boston University School of Medicine, Boston Medical Centre, Boston, MA 02118, USA; 6 Department of Neurology, The First Affiliated Hospital of Chongqing Medical University, Chongqing 40016, China; qili_md@126.com; 7 Section of Neuroradiology, Department of Experimental and Clinical Biomedical Sciences, University of Florence, 50134 Florence, Italy; henryfai@tin.it; 8 Department of Medicine (Neurology), Ottawa Hospital Research Institute, University of Ottawa, Ottawa, ON K1H 8M5, Canada; ddowlat@toh.ca; 9 Department of Emergency Medicine, Harvard Medical School, Massachusetts General Hospital, Boston, MA 02114, USA; jgoldstein@mgh.harvard.edu; 10 ASST Valcamonica, UOSD Neurology, Esine (BS), 25040 Brescia, Italy; andrea.morotti85@gmail.com

**Keywords:** intracerebral hemorrhage, imaging, outcome, ICH expansion, spot sign, NCCT markers

## Abstract

Intracerebral hemorrhage (ICH) accounts for 10% to 20% of all strokes worldwide and is associated with high morbidity and mortality. Neuroimaging is clinically important for the rapid diagnosis of ICH and underlying etiologies, but also for identification of ICH expansion, often as-sociated with an increased risk for poor outcome. In this context, rapid assessment of early hema-toma expansion risk is both an opportunity for therapeutic intervention and a potential hazard for hematoma evacuation surgery. In this review, we provide an overview of the current literature surrounding the use of multimodal neuroimaging of ICH for etiological diagnosis, prediction of early hematoma expansion, and prognostication of neurological outcome. Specifically, we discuss standard imaging using computed tomography, the value of different vascular imaging modalities to identify underlying causes and present recent advances in magnetic resonance imaging and computed tomography perfusion.

## 1. Introduction

Intracerebral hemorrhage (ICH) is one of the most devastating forms of stroke: one-year mortality approaches 50% and five-year survival is less than 30% [1]. While incidence has decreased over the past three decades, ICH was nevertheless responsible for 2.8 million deaths and 64.5 million disability-adjusted life years between 1990 and 2016 [2]. This significant global burden highlights an urgent need to develop novel strategies to treat and prevent ICH. There is currently no widely accepted therapeutic intervention for ICH. There are, however, numerous promising approaches being trialed for prevention, acute management and recovery, most of which rely on neuroimaging. Secondary prevention approaches range from hypertension control in small vessel vasculopathies to endovascular embolization of arteriovenous malformations; these are based on etiological diagnoses made with multimodal imaging. Similarly, emerging acute therapies require a rapid assessment of early hematoma expansion risk, which is both an opportunity for therapeutic intervention and a potential hazard for hematoma evacuation surgery. Prognostication and risk stratification are also intrinsically linked to neuroimaging features, including volume of hemorrhage, location of hemorrhage, and ventricular involvement. In this review, we provide an overview of the current literature surrounding the use of multimodal neuroimaging of ICH for etiological diagnosis, prediction of early hematoma expansion, and prognostication of outcome.

## 2. Imaging to Identify ICH Etiology

The term primary ICH traditionally refers to ICH arising from chronic, progressive cerebral small vessel disease, most commonly hypertensive arteriopathy (sporadic non-amyloid microangiopathies) and cerebral amyloid angiopathy (CAA) [3,4]. Conversely, secondary ICH refers to intracranial bleeding originating from either trauma (which is not included in this review) or intracranial pathology such as vascular malformations, tumor or other diseases. Table 1 gives an overview of common and uncommon causes of ICH and Table 2 summarizes red flags that should raise awareness for secondary ICH and suggests imaging modalities to investigate potential etiologies.

ICH location can provide important clues in the identification of ICH etiology. Deep ICHs are more commonly associated with long standing hypertension or other vascular risk factors whereas lobar bleedings are traditionally associated with CAA in the right clinical context. The CT features of ICH can also indicate the presence of CAA, with subarachnoid extension and finger-like projections being associated with higher odds of CAA [5], though these findings require external validation in real-life ICH cohorts.

Distinguishing CAA from hypertensive arteriopathy is important because patients with ICH due to CAA have a much higher risk of recurrence (8–10% vs. 1–2%) and have a higher risk of post-stroke dementia [6,7,8,9]. MRI markers of small vessel disease are more accurate than NCCT to distinguish hypertensive arteriopathy from CAA. In particular the strictly lobar distribution of multiple cerebral microbleeds and/or the presence of cortical superficial siderosis are strong validated markers of CAA included in the Boston Criteria. [10,11,12] Other CAA markers such as enlarged perivascular spaces and white matter hyperintensities extent have been described and may improve the diagnosis of CAA in clinical research settings and in clinical practice [13].

### 2.1. CTA and DSA for Macrovascular Causes

Following ICH detection on NCCT, intracranial vascular imaging, preferably computed tomographic angiography (CTA) should be acquired as it rapidly and accurately identifies several vascular abnormalities that require prompt intervention such as arteriovenous malformations (AVM) or aneurysms. Digital subtraction angiography (DSA) has a central role for further characterization of vascular anomalies, and to investigate ICHs with undetermined etiology, due to its higher spatial and temporal resolutions and consequent higher sensitivity for intracranial shunt detection that might be missed by CTA. The optimal timing for DSA is unknown, but should theoretically be performed as soon as possible to identify occult vascular malformations. Furthermore, therapeutic interventions can be performed in sequence at the time of the study with DSA if necessary. The angiographic yield of DSA, alone, is highest for younger patients (<45 years) without a history of hypertension, with absence of small vessel disease on NCCT, and with cerebellar or lobar ICH locations [14,15]. However, even in older adults with small vessel disease and pre-existing hypertension or deep ICH, DSA still detects other causes in ~2% [15]. In clinical routine, CTA has replaced DSA in many institutions as the initial vascular screening study because it is a non-invasive method that can detect macrovascular causes with good accuracy (95% sensitivity and 99% specificity) [16]. If CTA is negative DSA may still detect underlying causes in up to 20% if no small vessel disease or hypertension is known, suggesting that DSA should be performed if CTA is negative or inconclusive and should be considered for lower risk populations. Additionally, in presence of special characteristics such as calcification, subarachnoid hemorrhage or unusual hemorrhage location, DSA should be performed [17].

### 2.2. Value of CT-Perfusion

**Figure 1:**
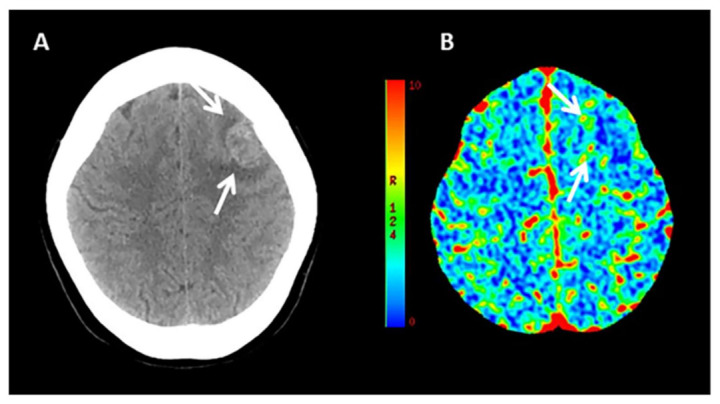
Example of CT Perfusion in a patient with ICH due to malignancy. Non-contrast CT (NCCT) scan (**A**) and cerebral blood volume (CBV) map (**B**) in a patient with acute ICH due to the bleeding of an underlying high grade glioma located left frontal lobe and characterized by elevated CBV values in the perihematomal region (arrows).

CT Perfusion (CTP) measures blood flow across brain capillaries (microcirculation) through the analysis of the first passage through cerebral vessels of an intravenous contrast bolus, using a tracer that remains intravascular since iodinated contrast does not cross the blood–brain barrier (BBB). CTP is a dynamic bolus-tracking technique based on repeated acquisition of a series of images before, during and after the injection of an intravenous contrast bolus. The role of CTP in identifying ICH etiology is currently very limited due to the obscuration of the underlying tissue by the extravasated blood leading to tissue destruction and anatomical distortion [18]. However, it has previously been demonstrated that peritumoral edematous CBV values are elevated in high grade gliomas and low in brain metastasis, suggesting the development of neoangiogenesis in the edema region surrounding high grade gliomas, related to infiltrative spreading of these lesions [19]. Therefore, as perihematomal edema is usually hypoperfused in spontaneous ICH [20], the occurrence of high perihematomal CBV levels can suggest that ICH is due to an underlying tumor (Figure 1) but this finding needs validation in larger studies. In addition, although CT Angiography can be considered the method of choice for rapid detection of ruptured vascular abnormalities [21], CTP pattern could better define the hemodynamic characteristics of any underlying brain arteriovenous malformation, providing different CTP profiles [22].

### 2.3. MRI

Additional diagnostic information about the underlying etiology may be obtained by magnetic resonance imaging (MRI) with contrast media. If clinical suspicion for an underlying neoplastic process is high, serial MRI with contrast may be performed once the hematoma has resolved to ensure that the hematoma does not obscure an underlying mass in the acute phase. Moreover, MRI may more strongly suggest the diagnosis of cerebral amyloid angiopathy (CAA) by the presence of numerous small foci of susceptibility blooming in the cerebral white matter on susceptibility weighted (SWI) sequences [23,24,25]. Recent studies also suggest an excellent sensitivity of MRI for the detection of arteriovenous shunting when using SWI and arterial spin label sequences, particularly in the detection and evaluation of dural arteriovenous fistulas (DAVF) [26,27].

## 3. Imaging to Predict Ich Expansion

ICH is a dynamic disease and up to half of ICH patients experience active bleeding leading to hematoma enlargement [28]. Hematoma Expansion (HE) is independently associated with unfavorable prognosis and represents therefore an appealing target for acute treatment [29]. Accurate stratification of ICH expansion risk is therefore highly desired, in order to identify patients at high risk of HE and therefore more likely to benefit from antiexpansion therapies [30].

The CTA spot sign, representing contrast extravasation on CT-angiography, is a robust and validated marker of HE with a sensitivity of 51% and a specificity of 88% [31] leading to some prediction tools incorporating this imaging variable [32,33]. The main limitation of this marker is that its evaluation relies on the acquisition of CTA, an imaging technique that is not widely available and in many centers is not routinely performed in the acute phase of ICH. In a large randomized controlled trial less than one in five patients received a CTA [34] and more recently two trials using the spot sign to target hemostatic treatment were terminated prematurely because of recruiting challenges, raising the need for a non-CTA dependent marker of HE [35].

**Figure 2:**
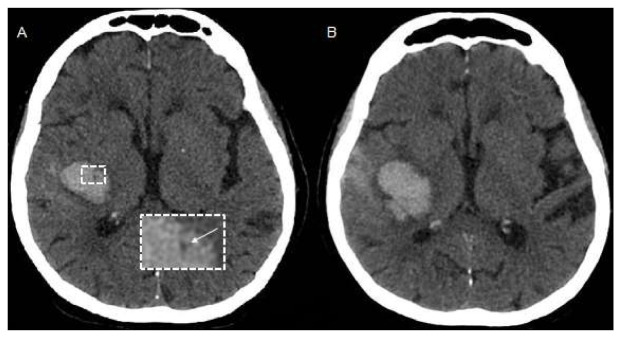

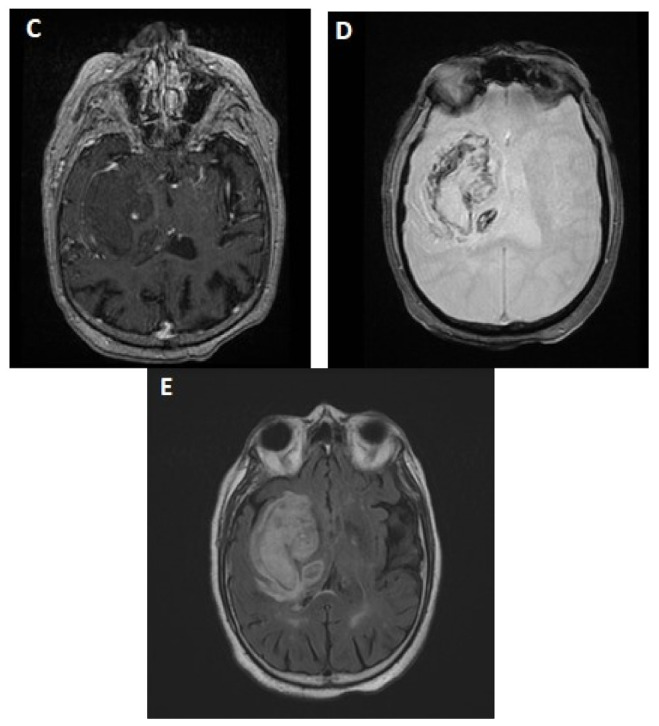
(**A**,**B**): Example of NCCT markers of ICH expansion. (**A**) Baseline NCCT with evidence of deep ICH and intrahematoma hypodensity (arrow), (**B**) Follow-up NCCT showing hematoma enlargement. (**C**–**E**) illustrate spot sign on T1-weighted post-contrast magnetic resonance imaging (MRI, **C**) within the ICH shown on susceptibility weighted images (SWI, **D**) and Fluid Attenuated Inversion Recovery (FLAIR) Sequence (**E**).

Multiple non-contrast CT (NCCT) ICH features have emerged as reliable alternative to the CTA spot sign [36] (Figure 2). NCCT markers can be categorized into ICH shape features and ICH density features and consensus criteria for their detection and interpretation have been recently proposed to harmonize terminology and use in future studies [37]. Of the reported markers, especially hypodensities within the hyperdense hematoma (Figure 2), the blend-sign and the black hole sign have been reported to show the highest sensitivity and specificity for prediction of ICH expansion and a good interrater reliability [37,38,39,40,41,42]. Of note, NCCT markers are not simply an epiphenomenon of the spot sign and if CTA images are available, use of both NCCT and CTA markers may provide additional yield in the stratification of HE risk [43].

Other imaging characteristics on NCCT may help the evaluation of HE probability. In particular, ICH volume is directly associated with the odds of hematoma enlargement whereas longer time from symptom onset to baseline imaging is inversely related with the risk of HE [44].

Several questions remain unanswered in the field of HE prediction with imaging. First, different definitions of the CTA spot sign have been reported and there are few data on a direct comparison between them [45]. Second, some NCCT markers still need prospective validation on a large sample size. Third, although a good inter-rater reliability have been recently shown [46], it remains to be determined whether this can be obtained also by investigators and clinicians with limited experience in acute neuroimaging of ICH. Finally, and most importantly, it remains unclear whether the use of NCCT and NCCT-based prediction tools can improve the discriminative ability of currently available prediction models.

### 3.1. MRI and ICH Expansion

**Figure 3:**
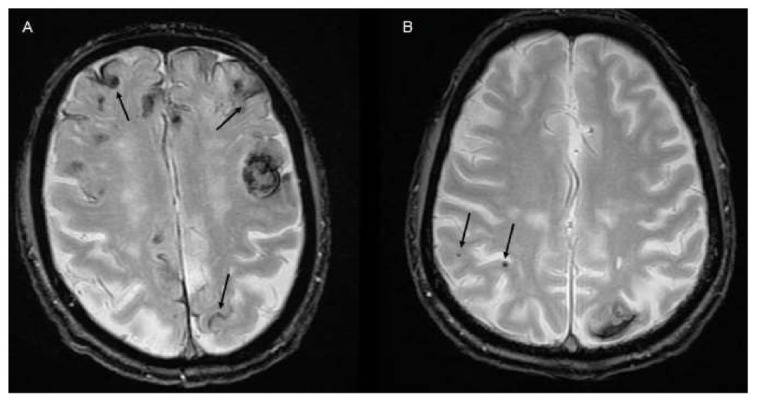
Small vessel disease markers. (**A**) MRI with evidence of lobar ICH and diffuse cortical superficial siderosis (arrows). (**B**) MRI with evidence of lobar ICH and lobar cerebral microbleeds (arrows).

The role of MRI in identifying patients at higher risk for HE remains poorly reported. Although speculative, the likeliest reason for the lack of data relative to the numerous reports on NCCT and CTA lies in the limited use of MRI at the hyperacute phase of stroke in many centers, making such data less generalizable and actionable to improve patients’ outcome. Still, several reports have examined the association between MRI biomarkers and ICH growth yielding to date two important contributions to ICH expansion research. First, fast-paced MRI sequences have allowed to visualize the dynamics of active intracerebral contrast leakage in MRI both in animal models [47], and anecdotally in human [48], providing pathophysiological insight into the phenomena underlying HE. Second, MRI allows to assess the association between pre-existing cerebral small vessel disease (cSVD) parenchymal damage, and the acute growth and final volume of ICH (Figure 3). Notably, the presence of cortical superficial siderosis as well as the absence of cerebral microbleeds have been associated with higher odds of HE and larger final ICH volumes in patients with primary ICH [49], suggesting that pre-existing microvessels’ frailty may contribute to the secondary shearing of surrounding microvessels and promote HE. Notably though, the treatment modifying effect of cSVD markers remains to be demonstrated and most of the studies in the field are retrospective and prone to convenience sample bias [50,51]. Finally, several reports and case series [52,53,54] of MRI “spot-signs” seen as acute intra-hemorrhage gadolinium extravasation on T1 weighted sequences have been published. The MRI spot sign has been consistently linked to poorer functional outcome, but data regarding HE remain limited [53].

### 3.2. Value of CT-Perfusion

Hemorrhagic transformation represents one of the most dangerous complication in patients with acute ischemic stroke treated with reperfusion therapies and it is associated with poor outcome. A relationship between very low CBV levels measured by CTP in ischemic area and risk of hemorrhagic transformation was reported in some studies, probably reflecting severe ischemia that promotes a damage of vascular integrity and, as a consequence, blood extravasation [55,56]. Based on these observations, the potential relationship between perihematomal CBV values and HE was recently investigated [57]. In a population of 155 patients with acute spontaneous ICH who underwent NCCT and CTP at admission and follow-up NCCT at 24 ± 6 h from baseline NCCT, perihematomal CBV values were inversely correlated with HE. More important, a decrease of perihematomal CBV levels was independently associated with HE, after adjustment for potential confounders. CBV stratification in quartiles revealed that HE was significantly related to very low perihematonal CBV values with a cut-off point<1.4 mL/100g representing the optimal CTP value to identify HE.

## 4. Imaging to Predict Outcome

Several neuroimaging characteristics can provide information on outcome, and accurate prognostic stratification is highly desired by patients, family members and clinical providers. The ICH score, one of the most commonly used prognostic tools, is heavily based on imaging items [58]. ICH volume is the strongest predictor of poor prognosis and can be rapidly estimated on baseline NCCT with the ABC/2 method [59]. Infratentorial location and presence of intraventricular hemorrhage are the other imaging items included in the ICH score, both associated with higher risk of poor prognosis such as mass effect and midline shift. Of note, the optimal timing for the use of prognostic tools remains unclear and recent evidence suggest that the use of follow-up rather than baseline ICH volume may improve the discriminative ability of the prognostic scores [60]. Multiple NCCT shape and density features have been linked with poor clinical evolution. [38,39,61] Recently a meta-analysis including more than 10,000 patients confirmed the prognostic significance of several NCCT markers, such as black hole sign, swirl sign, heterogeneous density, blend sign, hypodensities, irregular shape, and island sign [36]. When CTA images are available, the CTA spot sign is also independently associated with higher risk of death and unfavorable functional outcome [62]. From a pathophysiological point of view, the prognostic impact of NCCT markers and CTA spot sign is probably mediated by an increased probability of early clinical deterioration due to active bleeding and hematoma expansion. Finally, NCCT signs of cerebral small vessel disease such as leukoaraiosis and brain atrophy may help the identification of patients more likely to have a poor prognosis [63]. In conclusion, several imaging features are associated with the odds of death and severe disability after acute ICH. Most of the key outcome predictors can be evaluated on NCCT without advanced neuroimaging.

### 4.1. MRI

The role of MRI biomarkers is firmly established for the mid- and long-term prognostication of patients with ICH. MRI is central to understanding the underlying etiology with direct implications in terms of future therapeutic management and overall prognosis. MRI biomarkers of cSVD have, further, been shown to be strongly associated with functional and cognitive outcomes as well as hemorrhage recurrence risk and long-term mortality. As for HE prediction, MRI provides key information on pre-existing cSVD related brain damage. Accumulating evidence indeed links cerebral microbleeds [64], white matter injury, and other imaging markers of cSVD to poorer clinical outcome [64], higher risk for hemorrhage recurrence and increased mortality after an ICH. Cortical superficial siderosis has recently emerged as probably the strongest and most clinically relevant independent biomarker for poor prognosis, being associated with higher recurrence risk in patients with lobar ICH [8], worse cognitive trajectories and more frequent post-ICH dementia [65]. Altogether, acute MRI provides complementary information on ICH etiology, risk of recurrence, and likely clinical outcome [64].

### 4.2. CT-Perfusion

As stated before, it is well-known that the edematous region surrounding acute ICH is hypoperfused [20,66] More precisely, in the context of a concentrical distribution and a gradual improvement of perfusion parameters from the core to the periphery [20], no evidence for ischemic penumbra at risk for infarction was found in a previous CTP study demonstrating that perihematomal tissue is characterized by perfusion abnormalities indicative of oligoemia [20,66] and preserved autoregulation. Interestingly, perihematomal CBF and CBV values are lower in lobar than in deep ICH raising the hypothesis that small vessels included in perihematomal area of lobar ICH may be more susceptible to compression from edema mass effect [67]. Consistent with these results, the perihematomal area is currently considered as edematous tissue not at risk for infarction, evolving to spontaneous resolution [20,66]. As a consequence, the possible influence of perihematomal hemodynamic disturbances on outcome still remains poorly explored. However, in a previous Xenon-CT study [68], the predictive value of reduced perihemorrhagic CBF levels for in-hospital discharge status was documented in patients with subacute ICH. In addition, perihematomal CBF values were lower in patients with poor outcome (modified Rankin Scale 3–6) compared to those with good outcome (modified Rankin Scale 0–2) in a large population of patients with acute spontaneous ICH imaged at admission with NCCT and CTP. However, while no relationship was found between perihematomal CBF levels analyzed as continuous variables and outcome, only perihematomal CBF values < 40 mL/100 g/min were independently associated with unfavorable outcome. As HE is associated with poor outcome independently of perihematomal CBF values and perihematomal edema volume, the severity of hypoperfusion is another possible explanation for these findings. To summarize, these preliminary data suggest a potential role of perihematomal CTP values in clinical outcome stratification and warrant future confirmative studies.

## 5. Imaging Acute ICH in Clinical Practice

ICH is usually first detected on noncontrast computed tomography (NCCT) in an emergency setting. NCCT rapidly and accurately identifies an acute ICH, is less expensive than other imaging modalities and is widely available. For these reasons, NCCT is recommended as first-line imaging for diagnosing ICH in patients presenting with acute neurological deficits [69]. NCCT is also ideal for repeat imaging of ICH, for either routine monitoring or after neurological deterioration.

**Figure 4:**
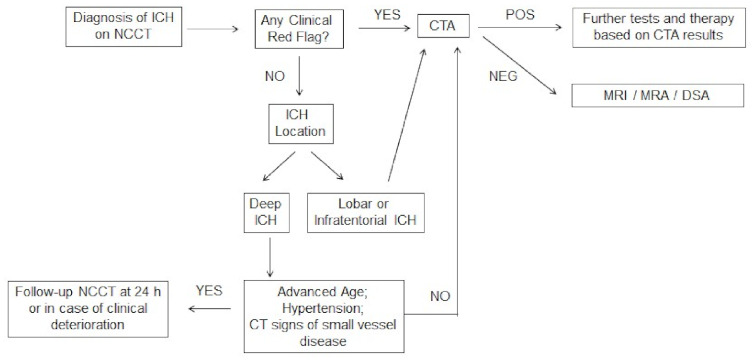
Flowchart for diagnostic work-up in patients with acute ICH.

Neuroimaging findings can provide important clues to identify the underlying ICH cause and the probability of an underlying vascular or other lesion. The neuroimaging workup should be guided by the clinical probability of a macrovascular lesion or other cause of secondary ICH [70]. The risk of secondary ICH is higher in patients with lobar or infratentorial ICH and in cases with isolated intraventricular hemorrhage and these patients should receive urgent CTA [15,71]. Conversely, the yield of CTA is probably very low in subjects with a deeply located bleeding, advanced age and presence of history of hypertension and CT markers of small vessel disease (leukoaraiosis). We suggest MRI/MRA and/or DSA in all patients without a clear cause of ICH after initial assessment with CTA. A pragmatic diagnostic algorithm for acute ICH imaging in clinical practice is provided in Figure 4.

## 6. Future Perspectives

Great potential for further enhancing optimal diagnosis of ICH and optimization of clinical workflows is in the use of machine-learning. A recent study found that an artificial intelligence algorithm was able to prioritize radiology worklists to reduce time to diagnosis of new outpatient ICH by 96% demonstrating the potential positive impact of advanced machine learning in radiology workflow optimization [72]. The same algorithm also identified subtle ICHs that were overlooked by radiologists further underlining the potential of machine-learning aided decision making in ICH diagnosis, especially in times of increasing imaging workloads [72]. Another group found that machine learning-based evaluation of image features provided the same discriminatory power in predicting functional outcome as multidimensional clinical scoring systems and that the integration of conventional scores and image features had synergistic effects with an increase of accuracy for prediction functional outcome in patients with ICH [73]. Further, mobile stroke units may not only help in the early diagnosis of ischemic stroke but also add to rapid diagnosis of ICH and to correctly triaging these patients to the appropriate hospital, which may especially be important in rural areas [74,75,76]. The use of advanced imaging techniques such as MR-spectroscopy promises to provide new insights into the diagnosis and pathophysiology of hemorrhage, the course of cerebral recovery, and the response to putative therapies not only of the ICH itself but also the surrounding edematous tissue [77,78].

## 7. Conclusions

This review gives an overview of the current literature surrounding the use of multimodal neuroimaging of ICH for etiological diagnosis, prediction of early hematoma expansion, and prognostication of neurological outcome. Specifically, it demonstrates standard imaging using computed tomography, discusses the value of different vascular imaging modalities to identify underlying causes and presents recent advances in magnetic resonance imaging and computed tomography perfusion.

## Figures and Tables

**Table 1:** Common and uncommon causes of ICH.

**Common causes**
Hypertension
Cerebral amyloid angiopathy
**Uncommon causes**
Arteriovenous malformations
Coagulopathy
Intracranial aneurysm
Cavernous angioma
Cerebral venous sinus thrombosis
Dural arteriovenous fistula
Moyamoya disease
Hemorrhagic transformation of ischemic infarct
Illicit drug abuse
Tumor
Vasculitis
Infective endocarditis
Posterior reversible encephalopathy syndrome

**Table 2:** Red flags for secondary ICH.

Possible Causes of Secondary ICH	Red Flags	Suggested Investigations
Arteriovenous malformation	young age; Lobar ICH;	MRI; CTA/MRA/DSA
Coagulopathy	History of anticoagulant use; Large and irregularly shaped hematoma	NCCT/CTA
Cavernous angioma	Recurrent ICH in the same location; Lobar or brainstem ICH	Hemorrhage surrounded by hypointense halo or rim on T2-weighted imaging; Punctuate hypointense foci on T2*GRE/SWI
Moyamoya disease	Onset of ICH at young age; Ischemic symptoms; Migraine-like headache;	MRI showing “ivy sign”-linear high signals; DSA showing typical “puff-of-smoke” appearance
Intracranial aneurysm	Associated Subarachnoid hemorrhage	CTA/MRA/DSA showing intracranial aneurysm
Venous sinus thrombosis	Young and middle-aged patients; Moderate to severe headache; Lobar ICH	MRI showing infarcts not restricted to typical arterial territories; CTV/DSA
Tumor	Lobar ICH; disproportionate edema; Mass effect	MRI showing mass lesion
Posterior reversible encephalopathy syndrome	Headache; Encephalopathy; Seizures; Visual disturbance; Lobar ICH	MRI abnormalities in posterior or watershed region
Infective endocarditis	Fever; Valvular regurgitation	MRI showing associated infarcts; Echocardiogram showing valvular vegetation

Abbreviation: MRI = magnetic resonance imaging, CTA = computed tomographic angiography, MRA = magnetic resonance angiography, DSA = digital subtraction angiography, GRE = gradient echo, SWI = susceptibility-weighted imaging, CTV = computed tomographic venography, EEG = electroencephalogram.
